# Registration on the Renal Transplantation Waiting List and Mortality on Dialysis: an Analysis of the French REIN Registry Using a Multi-state Model

**DOI:** 10.2188/jea.JE20130193

**Published:** 2015-02-05

**Authors:** Jean-Baptiste Beuscart, Dominique Pagniez, Eric Boulanger, Alain Duhamel

**Affiliations:** 1 Department of Biostatistics, EA2694, Lille School of Medicine, Lille2 University, Lille, France; 2 Nephrology Department, CHU Lille, France; 3 EA2693, Lille School of Medicine, Lille2 University, Lille, France

**Keywords:** survival analysis, multi-state model, renal dialysis, waiting list, kidney transplantation

## Abstract

**Background:**

Access to the renal transplantation (RT) waiting list depends on factors related to lower mortality rates and often occurs after dialysis initiation. The aim of the study was to use a flexible regression model to determine if registration on the RT waiting list is associated with mortality on dialysis, independent of the comorbidities associated with such registration.

**Methods:**

Data from the French REIN registry on 7138 incident hemodialysis (HD) patients were analyzed. A multi-state model including four states (‘HD, not wait-listed’, ‘HD, wait-listed’, ‘death’, and ‘RT’) was used to estimate the effect of being wait-listed on the probability of death.

**Results:**

During the study, 1392 (19.5%) patients were wait-listed. Of the 2954 deaths observed in the entire cohort during follow-up, 2921 (98.9%) were observed in the not wait-listed group compared with only 33 (1.1%) in the wait-listed group. In the multivariable analysis, the adjusted hazard ratio for death associated with non-registration on the waiting list was 3.52 (95% CI, 1.70–7.30). The risk factors for death identified for not wait-listed patients were not found to be significant risk factors for wait-listed patients, with the exception of age.

**Conclusions:**

The use of a multi-state model allowed a flexible analysis of mortality on dialysis. Patients who were not wait-listed had a much higher risk of death, regardless of co-morbidities associated with being wait-listed, and did not share the same risk factors of death as wait-listed patients. Registration on the waiting list should therefore be taken into account in survival analysis of patients on dialysis.

## INTRODUCTION

To be eligible for renal transplantation (RT), patients treated by dialysis must first be registered on an official RT waiting list. Access to such a registration has been described in several epidemiological studies as the end-result of a complex selection process depending on medical and non-medical determinants.^1^^–^^9^ Significant disparities have also been observed between countries, as availability of kidney donors and access to RT depend on the degree of economic development of the country, national health policies on RT, and cultural factors associated with organ donation and transplantation.^9^^,^^10^

Surprisingly, studies that analyze mortality of patients on dialysis do not currently take into account registration on the waiting list. However, patients registered on the RT waiting list are very different from patients who are not wait-listed for two main reasons. First, wait-listed patients are invariably younger and healthier than other dialysis patients.^1^^–^^9^ Second, wait-listed patients may benefit from an RT and stop dialysis, while patients who are not wait-listed remain on dialysis until death. In a study based on simulations, the probabilities of death during dialysis were systematically overestimated because wait-listed patients were given better survival probabilities but stopped dialysis early for RT.^11^ It therefore remains unclear whether wait-listed patients have longer survival than other dialysis patients in real life, irrespective of characteristics associated with placement on the waiting list.

Several important methodological issues affect the analysis of the relationship between registration on the waiting list and mortality on dialysis. First, registration on the RT waiting list is an event that usually occurs after the initiation of dialysis. Second, wait-listed patients may undergo an RT, which is a second event that may occur during the follow-up while on dialysis. Third, after RT, wait-listed patients are, by definition, no longer on dialysis and exposed to new specific risk factors for death, such as use of immunosuppressive drugs.^12^^–^^14^

A multi-state model is a stochastic process that at any time occupies one of a set of discrete states, which can be health conditions or disease stages. Use of a multi-state model has been shown to be a pertinent and accurate method of analyzing complex clinical issues with multiple outcomes.^15^^,^^16^ Such a model may be appropriate for describing and analyzing the complex relationships between covariates and the following states: ‘Hemodialysis (HD), wait-listed’, ‘HD, not wait-listed’, ‘death’, and ‘RT’.

The aim of the present study was to determine if registration on the RT waiting list is associated with mortality on dialysis, independently of the comorbidities associated with such registration. We identified HD patients from the French national Renal Epidemiology and Information Network (REIN) registry and used a multi-state model to analyze outcomes of placement on RT waiting list, death, or RT over a 4-year period.

## METHODS

### REIN registry

The REIN registry includes all incident patients treated for end-stage renal disease either by dialysis or RT in France. It was set up in 2002 to provide a tool for public health decision-making, evaluation, and research related to renal replacement therapies for end-stage renal disease. It relies on a patient database which is regionally and nationally maintained by a network of nephrologists, epidemiologists, and public health representatives. An ongoing registration process ensures that all dialysis and transplant patients are listed. Details about this registry have been published elsewhere.^17^

### Study population

Adult patients aged 18 years or older at initiation of dialysis who started HD between January 1, 2002, and December 31, 2006, were identified through the French REIN registry exclusively in the 12 regions equipped with software connected to the software currently used for the national RT waiting list. In the present study, HD patients were exclusively included, as they accounted for 93% of dialysis patients in France.

### Study design

Patients were followed up until the occurrence of death while on dialysis, RT, loss to follow-up, or the end of the study. Patients were identified at registration on the RT waiting list; removal from the waiting list was not taken into account in the analysis. When patients were registered, they were wait-listed patients until the end of the study, death, or RT. All patients were followed up for at least 2 years, as the cut-off date was December 31, 2008.

### Data collection

The following baseline characteristics were retrieved from the REIN registry: age, sex, height, weight, albumin levels at initiation of dialysis, modality of first dialysis, smoking habits, and presence or absence of selected co-morbidities (diabetes, chronic obstructive pulmonary disease, congestive heart failure, myocardial infarction, peripheral arterial disease, cerebrovascular disease, cirrhosis, amputation, inability to ambulate, or severe behavioral disorder). Data regarding registration on the waiting list were also collected. The etiology of renal disease was classified according to the REIN classification.^17^

### Ethics statement

Approvals from the National Commission on Informatics and Liberty and from the Advisory Committee on Information Processing in Material Research in the Field of Health were obtained through the national REIN registry. Each patient was petitioned for written informed consent at the time of inclusion in the REIN registry.

### Statistical methods

Baseline characteristics were presented in terms of mean and standard deviation (SD) for continuous variables and expressed as frequency and percentage for categorical variables. All statistical calculations were carried out using R Statistical Software (The R Project for Statistical Computing, Vienna, Austria), including the Survival, Mstate, and MICE packages.^21^^,^^22^^,^^27^

#### Multi-state model

**Figure 1.**
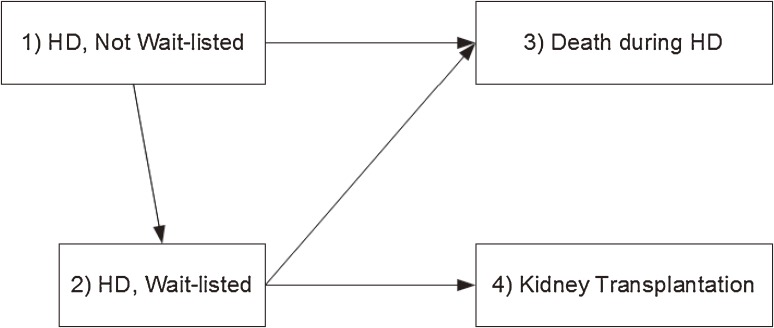
Multi-state model used in the study. All patients start treatment on hemodialysis (HD), and are considered as not wait-listed. Then they may die during HD (transition 1 → 3) or be put on the waiting list (transition 1 → 2). Once on the waiting list, patients may die during HD (transition 2 → 3) or undergo a renal transplantation (RT, transition 2 → 4). Follow-up stops after RT because patients are no longer on dialysis, and the risk factors for death are therefore different from those for dialysis patients.

A multi-state model including the four following states was applied: 1) ‘HD, not wait-listed’, ie, HD patients who were not registered on the RT waiting list; 2) ‘HD, wait-listed’, ie, HD patients who were registered on the RT waiting list; 3) ‘death’; and 4) ‘kidney transplantation’. Usually, a multi-state process is assumed to be a time-inhomogeneous Markov process^18^; this means that the future state of the process only depends on the current state and the elapsed time since the time of origin. As this model required the presence of a unique initial state, all patients were considered as ‘HD, not wait-listed’ at the onset; for patients who were already wait-listed at the initiation of HD, registration was set at day one. Death and RT were absorbent states, while being wait-listed was a transient state. Consequently, four transitions were possible, which are detailed in Figure 1.

The probabilities of being in each state at each time point of follow-up were estimated by the Aalen-Johansen estimator^19^ and were stacked for graphic presentation. As recommended, the duration shown on curves was 4 years, because only 10% of the patients were still under follow-up at the time “4 years”.^20^

The Cox proportional hazards regression model was used to investigate the influence of demographic, biological, and clinical factors on the transition from one given state to another. In each transition, this model provided a hazard ratio (HR) for each covariate, which was assessed by bi- and multi-variable analyses.^19^ The main objective of the present study was to estimate the influence of being wait-listed on the risk of death during HD. This corresponded to estimating the HR for transition ‘HD, not wait-listed’ → ‘death’ versus that for ‘HD, wait-listed’ → ‘death’. To do this, a transition-specific model was used, which was similar to considering the ‘HD, wait-listed’ state as a time-dependent covariate.^19^^,^^21^

#### Management of missing data

In the extracted data, 17 covariates had missing values, as shown in Table 1. Values for covariates with missing values were obtained by multiple imputations using the MICE package, as recommended for Cox proportional hazards model analysis.^22^ Regression switching imputation was performed using linear or logistic regression models, depending on the nature of the incomplete covariate fitted.^22^^,^^23^ This procedure was repeated five times to obtain five draws for each missing value in five distinct datasets.

**Table 1.** Baseline characteristics of the study population (*n* = 7138)

Characteristics	Patients (*n* = 7138)	Missing data (%)
Age^a^, years [mean (SD)]	67.5 (14.9)	0
Women, *n* (%)	2659 (37.3%)	0
Body mass index^a^, kg/m^2^ [mean (SD)]	25.2 (5.3)	26.2
Albumin^a^, g/l [mean (SD)]	33.7 (5.9)	54.3
Unplanned first dialysis, *n* (%)	2205 (31.0%)	0.6
Dialysis on catheter, *n* (%)	3119 (43.9%)	1.8
Smoking habits, *n* (%)		
Non smoker	4005 (63.8%)	11.7
Former smoker	1604 (25.6%)	11.7
Current smoker	666 (10.6%)	11.7
Selected co-morbidities^b^, *n* (%)		
Diabetes	2313 (35.6%)	8.6
Chronic obstructive pulmonary disease	726 (11.2%)	8.6
Congestive heart failure	1760 (27.1%)	8.6
Myocardial infarction	747 (11.5%)	8.7
Peripheral arterial disease	1494 (23.1%)	9
Cerebrovascular disease	639 (9.8%)	8.6
Cirrhosis	142 (2.2%)	8.9
Amputation	155 (2.2%)	1.5
Inability to ambulate	1325 (21.3%)	12.3
Severe behavioral disorder	249 (3.5%)	1.3
Primary renal disease, *n* (%)		
High blood pressure	1645 (23.0%)	0
Diabetes	1508 (21.1%)	0
Glomerulonephritis	810 (11.3%)	0
Pyelonephritis	311 (4.4%)	0
Polycystic kidney disease	491 (6.9%)	0
Vascular	129 (1.8%)	0
Other	1161 (16.3%)	0
Unknown	1083 (15.2%)	0

SD, standard deviation.

^a^Expressed as mean (SD).

^b^Co-morbidities examined in this study and listed for data collection.

In the multivariable analysis, covariates were selected using a stepwise procedure adapted to multiple imputation methodology.^24^ The covariates selected could vary for each transition. For an easier interpretation of the results, when a covariate was selected for a transition from a given initial state, this covariate was included in the two transitions relating to this initial state. Rubin’s approach was adopted, whereby the coefficients and variances obtained with the final model on each imputed dataset were averaged by taking into account the intra-variance of the model and the inter-variance between the imputed datasets.^25^

#### Sensitivity analysis

A sensitivity analysis was conducted to investigate the possible interaction between age and waiting list registration for the risk of death. The whole analysis strategy (ie, multi-state model analysis and management of missing data) was performed among five age groups: 18 to 39 years, 40 to 49 years, 50 to 59 years, 60 to 69 years, and 70 years and older.

#### Log-linearity assumption

The log-linear assumption of the Cox model was assessed using Martingale residuals.^26^ Since the log-linearity assumption was violated for age and body mass index (BMI), these variables were transformed into categorical variables. The scatter plots of Martingale residuals are presented as supplementary data in eFigure 1. The cut-off values were identified first by graphic investigations using Martingale residual plots, then by maximization of the Gray test, and finally on the basis of medical expertise and consensus.

## RESULTS

### Patients

We identified 7138 patients starting HD as first renal replacement therapy between January 1, 2002, and December 31, 2006. Their baseline characteristics are detailed in Table 1. A total of 176 (2.5%) patients were already wait-listed at the time of HD initiation, and 1392 patients (19.5%) were wait-listed at the cut-off date. This corresponded to 13 210 person-years observed in the not wait-listed group, and 2907 person-years observed in the wait-listed group, 1552 of which was on the waiting-list. The baseline characteristics of the patients according to the registration on the RT waiting list, as observed at the end of the study, are detailed as supplementary data in eTable 1.

### Likelihood of events

**Figure 2.**
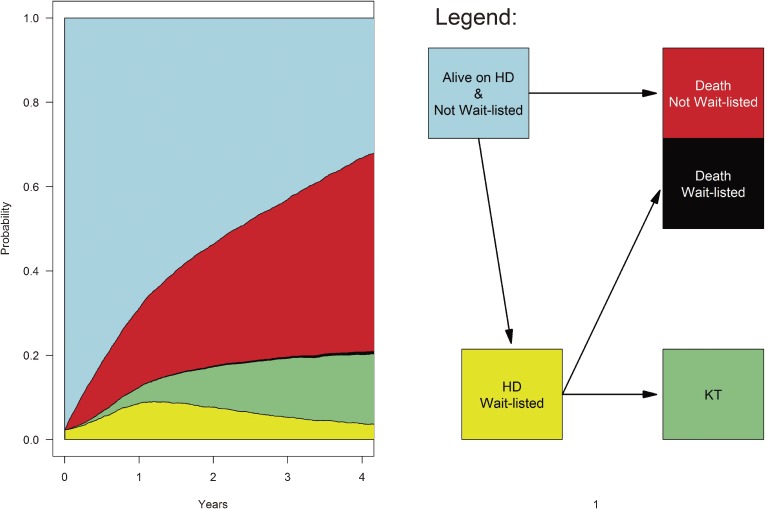
Probabilities of being in a given state at each follow-up time point, estimated by the Aalen-Johansen estimator. Probabilities are stacked. For example, the 2-year probabilities were estimated at 7.7% for the waiting list (yellow), 9.6% for renal transplantation (RT, green), 0.2% for death while on the waiting list (black), 28.9% for death while not wait-listed (red), and 53.6% for being alive on HD and not wait-listed (blue). The sum of these probabilities is 1.00 at each time point.

In the multi-state model, the initial state of all the patients corresponded to the ‘HD, not wait-listed’ state. The probabilities of being in a given state at each follow-up time point, as estimated by the Aalen-Johansen estimator, are shown in Figure 2. The probability of remaining not wait-listed and alive on HD was only 33.2% four years after the initiation of HD; the probability of remaining not wait-listed and dying was estimated at 46.0% (Figure 2). Thus, patients who remained not wait-listed had a very high probability of death on HD.

The probability of being wait-listed progressed in two stages, with an increase in the first year followed by a constant and regular decline, as illustrated in Figure 2. The registration rate was therefore higher at the onset, then subsequently lower, than the rate of RT. Most of the wait-listed patients were registered on the RT waiting list in the first year after HD initiation, with a median time before registration of 0.8 years (inter-quartile range, 0.4 to 1.4 years). The probability of undergoing RT increased rapidly after the first year and was estimated at 16.4% at 4 years, as shown in Figure 2. Conversely, the probability of death while being wait-listed was very low (estimated at 0.6% at 4 years).

These results show that patients who remained not wait-listed had a much higher probability of death than wait-listed patients. Of the 2954 deaths observed in the entire cohort during follow-up, 2921 (98.9%) were observed in the not wait-listed group compared with only 33 (1.1%) in the wait-listed group.

### Factors associated with registration on the RT waiting list

Using bivariate analysis, all co-morbidities were found to be significantly associated with registration on the waiting list and death while on HD (data not shown). The results of the multivariable analysis are presented in Table 2. All selected covariates except for gender were significant contraindications for registration on the waiting list (transition ‘HD, not wait-listed’ → ‘HD, wait-listed’). Notably, patients older than 70 years had an 80-fold (1/0.0126) lower probability of being wait-listed than patients under 50 years of age. Conversely, all selected co-morbidities except for diabetes, myocardial infarction, and stroke were significant risk factors for death on dialysis (transition ‘HD, not wait-listed’ → ‘death’), as shown in Table 2.

**Table 2.** Hazard ratio for transition 1 → 2 (Not wait-listed → Wait-listed) and transition 1 → 4 (Not wait-listed → Death during dialysis) in the multi-state model

	Transition 1 → 2 Not wait-listed → Wait-listed		Transition 1 → 4 Not wait-listed → Death	
	
	HR	95% CI	HR	95% CI
Age (years)				
<50	1.00		1.00	
50–55	0.64	(0.55, 0.75)	1.41	(1.02, 1.95)
55–60	0.54	(0.46, 0.63)	1.92	(1.45, 2.53)
60–65	0.31	(0.26, 0.37)	2.04	(1.58, 2.65)
65–70	0.14	(0.11, 0.18)	2.26	(1.77, 2.88)
>70	0.01^a^	(0.01, 0.02)	3.77	(3.03, 4.70)
Gender: female	0.92	(0.82, 1.03)	0.82	(0.76, 0.89)
BMI (kg/m^2^)				
22–30	1.00		1.00	
<22	0.84	(0.74, 0.95)	1.25	(1.14, 1.37)
>30	0.68	(0.57, 0.81)	0.91	(0.80, 1.02)
Albumin (increase of 1 g/l)	1.02	(1,01, 1.04)	0.99	(0.98, 0,99)
Dialysis on catheter	0.71	(0.63, 0.81)	1.37	(1.27, 1.49)
Selected co-morbidities^b^				
Diabetes	0.69	(0.54, 0.88)	1.10	(0.99, 1.23)
Chronic obstructive pulmonary disease	0.58	(0.43, 0.78)	1.20	(1.08, 1.33)
Congestive heart failure	0.61	(0.49, 0.75)	1.27	(1.17, 1.39)
Myocardial infarction	0.66	(0.48, 0.91)	1.10	(0.98, 1.22)
Peripheral arterial disease	0.60	(0.47, 0.77)	1.14	(1.04, 1.25)
Cerebrovascular disease	0.65	(0.48, 0.87)	1.12	(1.00, 1.26)
Cirrhosis	0.25	(0.11, 0.61)	1.78	(1.43, 2.22)
Inability to ambulate	0.35	(0.23, 0.54)	1.77	(1.62, 1.95)
Severe behavioral disorder	0.27	(0.16, 0.47)	1.52	(1.28, 1.81)
Primary renal disease				
Polycystic kidney disease	1.00		1.00	
High blood pressure	0.57	(0.46, 0.71)	1.35	(1.06, 1.71)
Diabetes	0.55	(0.41, 0.75)	1.49	(1.15, 1.94)
Glomerulonephritis	1.08	(0.92, 1.27)	1.19	(0.91, 1.56)
Pyelonephritis	0.65	(0.50, 0.84)	1.49	(1.11, 2.00)
Vascular	0.57	(0.32, 0.99)	1.62	(1.17, 2.24)
Other	0.56	(0.46, 0.68)	1.98	(1.55, 2.52)
Unknown	0.61	(0.50, 0.75)	1.62	(1.26, 2.07)

BMI, body mass index; CI, confidence interval; HR, hazard ratio.

^a^Estimated HR amounted to 0.012 600 21.

^b^Co-morbidities listed for data collection.

Patients who remained not wait-listed tended to be significantly older and to have more co-morbidities; these characteristics significantly increased their probability of death.

### Hazard ratio for death associated with registration on the RT waiting list

The comparison between the risk of death for wait-listed patients and patients who were not wait-listed was carried out by means of the transition-specific model. The results are presented in Table 3. In bivariate analysis, patients who were not wait-listed displayed a greater risk of death than wait-listed patients (HR 8.83; 95% CI, 6.26–12.44). The adjusted HR for death associated with not being wait-listed was 3.52 (95% CI, 1.70–7.30). Remaining not wait-listed significantly increased the risk of death while on HD, regardless of the impact of co-morbidities on the probability of being wait-listed. Sensitivity analysis showed that the results were similar for death associated with being not wait-listed between the different age groups. Results are displayed as supplementary data in eTable 2.

**Table 3.** Hazard ratio for death associated with being not wait-listed, estimated using the transition-specific model

	HR^a^	95% CI
Unadjusted	8.83	(6.26, 12.44)
Adjusted on age and co-morbidities^b^	3.52	(1.70, 7.30)

CI, confidence interval; HR, hazard ratio.

^a^The HR describes the ratio for the transition hazard 1 → 4 (Not wait-listed → Death during dialysis) and the transition hazard 2 → 4 (Wait-listed → Death during dialysis).

^b^Adjusted for age, sex, BMI, albumin level, dialysis on catheter, diabetes, chronic obstructive pulmonary disease, congestive heart failure, myocardial infarction, peripheral arterial disease, cerebrovascular disease, cirrhosis, amputation, inability to ambulate, severe behavioral disorder, and primary renal disease.

### Factors associated with death and RT after registration

In the multi-state model analysis, the factors associated with RT (transition ‘HD, wait-listed’ → ‘RT’) and death of the wait-listed patients (transition ‘HD, wait-listed’ → ‘death’) were investigated. The results of the multivariate analysis are presented in Table 4. It was found that only age over 60 years and inability to ambulate significantly increased the probability of receiving a transplant, and only age over 60 years and a history of myocardial infarction were significant risk factors for death. These results show that very few comorbidities influenced the outcome of patients following registration on the waiting list.

**Table 4.** Hazard ratio for transition 2 → 4 (Wait-listed → Death during dialysis) and 2 → 3 (Wait-listed → Renal transplantation [RT]) in the multi-state model

	Transition 2 → 4 (Wait-listed → Death)		Transition 2 → 3 (Wait-listed → RT)	
	
	HR	95% CI	HR	95% CI
Age (years)				
<60	1.00		1.00	
>60	2.49	(1.20, 5.15)	1.26	(1.08, 1.46)
Female sex	1.97	(0.98, 3.98)	0.99	(0.87, 1.13)
Myocardial infarction	6.46	(2.24, 18.65)	1.05	(0.65, 1.70)
Inability to ambulate	—^a^	—	1.64	(1.06, 2.54)

CI, confidence interval; HR, hazard ratio.

^a^HR could not be estimated because no death events were observed for waiting-list patients with an inability to ambulate.

## DISCUSSION

In the present study, we identified an independent relationship between registration on the RT waiting list and the probability of death while on dialysis. Patients who remained not wait-listed were 8.83 times more likely to die while on dialysis than wait-listed patients during follow-up. This HR for death was explained in part by the fact that age and co-morbidities were both significant factors that constituted contraindications for registration on the waiting list. However, even after adjustment for age and co-morbidities, patients who were not wait-listed were still 3.52 times more likely to die while on dialysis than wait-listed patients.

Our large cohort of incident HD patients identified through the national REIN registry in regions connected to the national information system of the French Transplantation Agency makes our findings especially noteworthy. We showed that adjustment for baseline characteristics in a classic multivariate model is not enough to account for the association with mortality and registration on the RT waiting list. Registration on the RT waiting list is the result of a complex decision-making process based on medical expertise in accordance with medical guidelines.^1^^,^^28^^,^^29^ The adjusted HR for death associated with being not wait-listed highlights that this medical expertise provides additional and detailed information on the probability of death while on dialysis, regardless of co-morbidities.

Registration on the waiting list is an administrative event. Neither the health status of patients nor the dialysis treatment status change at the date of registration. Therefore, registration cannot in itself modify the probability of death of a patient, like a myocardial infarction or chemotherapy would do. The high hazard of death associated with being not wait-listed may reflect the effect of numerous other risk factors for death in wait-listed patients, like socio-economic and psychological characteristics, treatment adherence, and miscellaneous diseases. Such risk factors are usually not reported in registries or studies on mortality in dialysis. Consequently, registration on the waiting list appears as an essential adjustment factor in the survival analysis of dialysis patients.

It is well-recognized that co-morbid conditions strongly and independently limit access to the RT waiting list, especially diabetes in elderly patients.^8^ Thus, dialysis patients have been shown to have worse co-morbid conditions than transplant recipient.^5^ In the present study, we have clarified such findings; the multi-state model analysis allowed us to show simultaneously that all co-morbidities examined in this study, except history of diabetes, myocardial infarction, and stroke, were contraindications to placement on the RT waiting list and risk factors for death while on dialysis. In particular, elderly patients over 70 years were 80 times less likely to be wait-listed than patients under 50 years, whereas their risk of death was only 3.7 times greater than patients under 50 years. Our results are consistent with previous reports that elderly patients are less likely to be wait-listed than their younger counterparts, but we found a greater risk of not being wait-listed among elderly patients than previously published studies.^5^^,^^7^^,^^8^^,^^10^ Consequently, elderly patients over 70 years had a lower chance of being evaluated for RT in France, regardless of their health status.

Using a multi-state model, we have shown that most of the risk factors for death identified in patients who were not wait-listed (Table 2) were different from those identified in wait-listed patients (Table 4). This finding suggests that failure to consider registration on the RT waiting list in outcome analysis could lead to incomplete or erroneous interpretation of data. Let us suppose that *A* is a risk factor for death in patients who were not wait-listed, but not in wait-listed patients; a study not taking into account placement on the waiting list when analyzing data could wrongly conclude that *A* is a risk factor for death in all HD patients, including wait-listed patients. Given such a hypothesis, wait-listed patients may receive unnecessary medical treatment to correct *A*, which could be a source of potential error, adverse effects, and unnecessary expense.

Registration on the waiting list, a time-dependent event, was examined using a multi-state model. Considering registration as a baseline covariate, regardless of the time spent on HD before registration, would cause a bias known as immortality bias.^30^^,^^31^ A classical survival analysis with a time-dependent covariate would not take into account the competing risk of RT. The multi-state model corresponds to the general framework of competing risks.^18^ The use of this model in the present study showed that it is well suited to assessing the effect of registration on the waiting list, an intermediate and time-dependent state, on the outcome of patients on HD.

Our results were obtained on the basis of data extracted from a national registry, which allowed adjustments to be made for a number of covariates on a large number of patients. The information regarding registration on the waiting list was accurate. Therefore, given the size of the study cohort analyzed and the statistical model used, the present results appear reliable.

Several limitations, however, must be considered in interpreting our findings. First, the structure for allocating organs, the decision-making process, and the waiting time before registration and RT vary between countries,^3^^,^^9^^,^^10^^,^^32^ so the present results may be specific to France. However, low death rates for patients on RT waiting lists have also been reported in other western countries.^33^^–^^35^ Although the low death rates reported among other studies of RT waiting lists suggest a similar effect of registration on the waiting list on the probability of death, this apparent trend should be confirmed by further studies in other countries. Second, all factors associated with registration on the waiting list, as reported in the literature, may not have been fully adjusted for. However, as registration is a dynamic process that does not depend only on co-morbidities at baseline, it would be extremely difficult to take into account all factors that could influence waiting list registration. Finally, statistical analysis is underpowered for the analysis of risk factors for death among wait-listed patients due to the low number of events (only 33 deaths were observed in this patient group). However, the majority of wait-listed patients underwent RT, and death while on dialysis was not observed in these patients. When conducting a multivariate survival analysis, it is recommended to have at least 10 events observed for each covariate.^36^ According to the incidence of death events and registration on the waiting list among wait-listed patients in our study, it would be necessary to include at least 20 000 HD patients to study 10 covariates in the wait-listed group, which is impractical.

### Conclusion

In conclusion, this study demonstrated that registration on the RT waiting list should be considered in the survival analysis of patients on dialysis. Indeed, registration on the waiting list appeared to be a selection process leading patients with a worse prognosis to remain on dialysis and those with a better prognosis to undergo RT. Further, wait-listed patients and patients who were not wait-listed did not share the same risk factors for death, but the interpretation of our result was limited by a lack of power. Further studies of larger cohorts are needed to confirm these preliminary data. Finally, our findings suggest that using a multi-state model and considering registration on the RT waiting list as an intermediate state may avoid misinterpretation of the risk factors for death.

## ONLINE ONLY MATERIALS

eTable 1. Baseline characteristics of patients according to registration on the renal transplantation waiting list. Comparisons between the two groups of patients were not performed because the groups were constituted during the study, not at baseline.

eTable 2. Hazard ratio for death associated with not being wait-listed by age groups, estimated by the transition-specific model.

eFigure 1. Scatter plots of Martingale residuals for age and body mass index for each transition of the multi-state model used in the study.
