# Supplementary material for: Registration on the Renal Transplantation Waiting List and Mortality on Dialysis: an Analysis of the French REIN Registry Using a Multi-state Model
Source: J Epidemiol. 2015 Feb 5;25(2):133–41. doi: 10.2188/jea.JE20130193 (PMC4310874; doi:10.2188/jea.JE20130193)
Supplement: eTable 1. [file je-25-133-s001.pdf]

eTable 1. Baseline characteristics of patients according to registration on the renal transplantation waiting list. Comparisons between the two groups of patients were not performed because the groups were constituted during the study, not at baseline.

**Characteristics**	**Wait-listed patients (n=1,403)**	**Not-wait-listed Patients (n=5,735)**
Age[(a)], years [mean (SD)]	49.1 (12.9)	72.0 (11.5)
Women, n (%)	494 (35.2%)	2,165 (37.8%)
Body mass index[(a)], kg/m² [mean (SD)]	24.6 (4.7)	25.4 (5.4)
Albumin[(a)], g/l [mean (SD)]	35.9 (5.7)	33.1 (5.9)
Unplanned first dialysis, n (%)	303 (21.6%)	1,902 (33.4%)
Dialysis on catheter, n (%)	422 (30.1%)	2,697 (47.2%)
Smoking habits, n (%)		
Non-smoker	707 (60%)	3,298 (64.7%)
Former smoker	263 (22.3%)	1,341 (26.3%)
Current smoker	208 (17.7%)	458 (9%)
Selected co-morbidities[(b)], n (%)		
Diabetes	211 (17.3%)	2,102 (39.9%)
Chronic obstructive pulmonary disease	42 (3.4%)	684 (13%)
Congestive heart failure	88 (7.2%)	1,672 (31.8%)
Myocardial infarction	39 (3.2%)	708 (13.5%)
Peripheral arterial disease	67 (5.5%)	1,427 (27.2%)
Cerebrovascular disease	44 (3.6%)	595 (11.3%)
Cirrhosis	7 (0.6%)	135 (2.6%)
Amputation	4 (0.3%)	151 (2.7%)
Inability to ambulate	23 (1.8%)	1,302 (26.2%)
Severe behavioral disorder	14 (1%)	235 (4.2%)
Primary renal disease, n (%)		
High blood pressure	141 (10%)	1,504 (26.2%)
Diabetes	144 (10.3%)	1,364 (23.8%)
Glomerulonephritis	366 (26.1%)	444 (7.7%)
Pyelonephritis	79 (5.6%)	232 (4%)
Polycystic kidney disease	265 (18.9%)	226 (3.9%)
Vascular	13 (0.9%)	116 (2%)
Other	226 (16.1%)	935 (16.3%)
Unknown	169 (12%)	914 (15.9%)

SD, standard deviation.
