# Supplementary material for: Registration on the Renal Transplantation Waiting List and Mortality on Dialysis: an Analysis of the French REIN Registry Using a Multi-state Model
Source: J Epidemiol. 2015 Feb 5;25(2):133–41. doi: 10.2188/jea.JE20130193 (PMC4310874; doi:10.2188/jea.JE20130193)
Supplement: eTable 2. [file je-25-133-s002.pdf]

eTable 2. Hazard ratio for death associated with not being wait-listed by age groups, estimated by the transition-specific model

	Aged 18 to 39 years		Aged 40 to 59 years		Aged 50 to 59 years		Aged 60 to 69 years[(c)]	
	HR[(a)]	95% CI	HR[(a)]	95% CI	HR[(a)]	95% CI	HR[(a)]	95% CI
Unadjusted	4.59	(1.30, 16.17)	4.44	(2.22, 8.87)	5.95	(3.03, 11.69)	3.72	(1.96, 7.06)
Adjusted model [(b)]	3.75	(0.99, 14.24)	2.77	(1.35, 5.69)	4.07	(2.04, 8.12)	3.97	(1.99, 7.94)

CI, confidence interval; HR, hazard ratio.

[a] The HR describes the ratio for the transition hazard 1→4 (Not wait-listed → Death during dialysis) and the transition hazard 2→4 (Wait-listed → Death during dialysis).

[b] Adjusted according to the selected variables for each age class:

- 18-39 years: sex, BMI, dialysis on catheter, diabetes, chronic obstructive pulmonary disease, congestive heart failure, cirrhosis, and inability to ambulate
- 40-49 years: albumin level, dialysis on catheter, peripheral arterial disease, cirrhosis, severe behavioral disorder, and primary renal disease
- 50-59 years: dialysis on catheter, congestive heart failure, myocardial infarction, peripheral arterial disease, cirrhosis, inability to ambulate, severe behavioral disorder, and primary renal disease
- 60-69 years and 18-69 years: sex, BMI, albumin level, dialysis on catheter, diabetes, chronic obstructive pulmonary disease, congestive heart failure, myocardial infarction, peripheral arterial disease, cerebro-vascular disease, cirrhosis, inability to ambulate, severe behavioral disorder, and primary renal disease

[c] The analysis could not be performed for patients aged 70 years and older because too few death events were observed in the wait-listed group among these patients.
