# Supplementary material for: Registration on the Renal Transplantation Waiting List and Mortality on Dialysis: an Analysis of the French REIN Registry Using a Multi-state Model
Source: J Epidemiol. 2015 Feb 5;25(2):133–41. doi: 10.2188/jea.JE20130193 (PMC4310874; doi:10.2188/jea.JE20130193)

e-Figure 1. Scatter plots of Martingale residuals for age and body mass index for each transition of the multi-state model used in the study

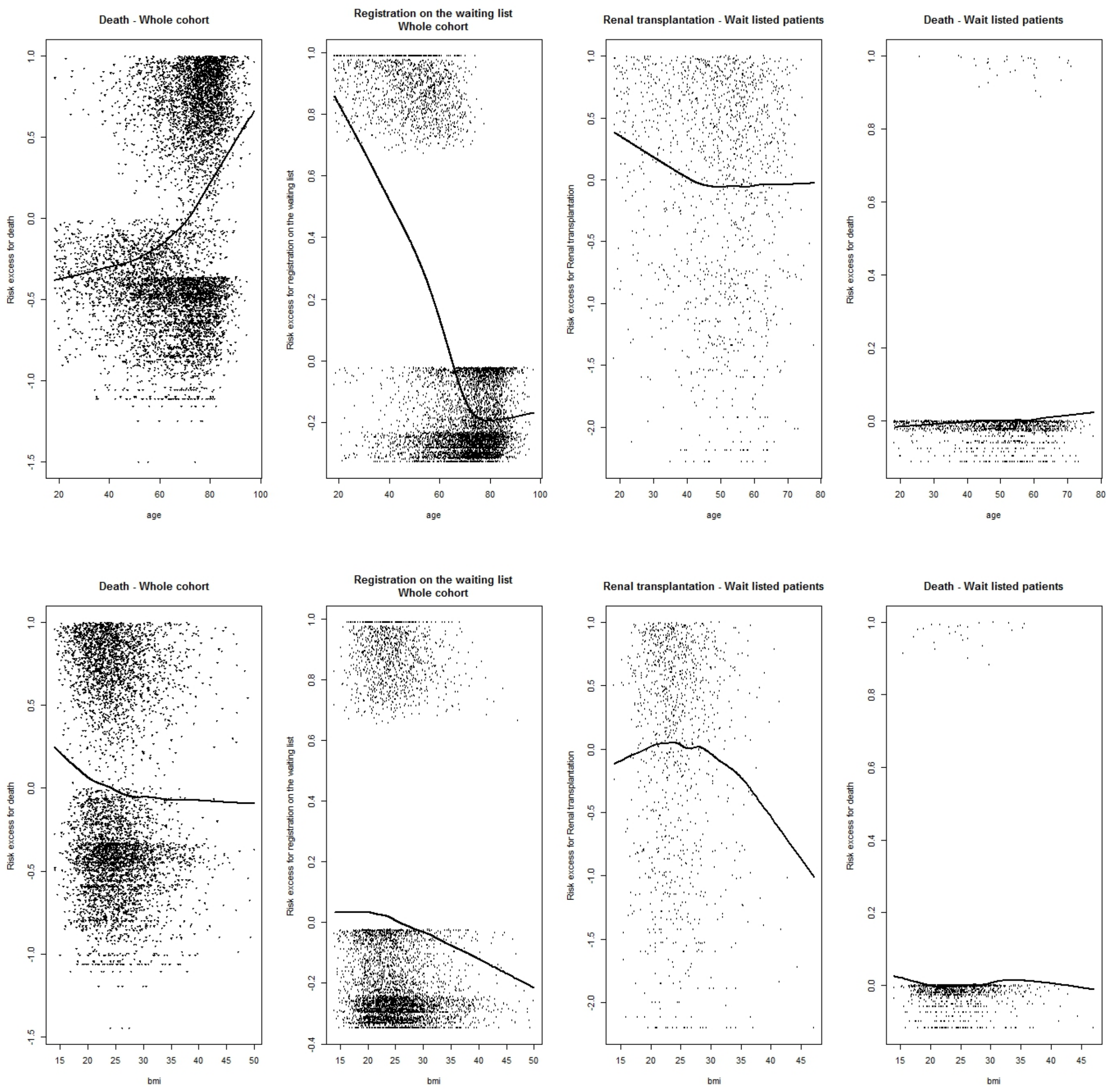

Supplement: eFigure 1. [file je-25-133-s003.pdf]
